# Concurrent Influenza and Shigellosis Outbreaks, Papua New Guinea, 2009

**DOI:** 10.3201/eid1704.101021

**Published:** 2011-04

**Authors:** Alexander Rosewell, Rosheila Dagina, Manoj Murhekar, Berry Ropa, Enoch Posanai, Samir Dutta, Ian Barr, Glen Mola, Anthony Zwi, C. Raina MacIntyre

**Affiliations:** Author affiliations: World Health Organization, Port Moresby, Papua New Guinea (A. Rosewell, M. Murhekar);; University of New South Wales, Sydney, New South Wales, Australia (A. Rosewell, A. Zwi, C.R. MacIntyre);; National Department of Health, Port Moresby (R. Dagina, B. Ropa, E. Posanai);; Port Moresby General Hospital, Port Moresby (S. Dutta);; World Health Organization Collaborating Center for Reference and Research on Influenza, Melbourne, Victoria, Australia (I. Barr);; University of Papua New Guinea, Port Moresby (G. Mola)

**Keywords:** Influenza, Shigella, shigellosis, viruses, bacteria, outbreak, Papua New Guinea, letter

**To the Editor:** A high case-fatality ratio has often been associated with outbreaks of a new influenza virus but is less commonly reported in association with seasonal influenza. Nevertheless, in developing countries, seasonal influenza has been associated with a high proportion of deaths, especially among remote populations. In Madagascar, seasonal influenza mortality rates of 2.5% have been reported (*1*), with even higher rates (15%) reported in Indonesia (*2*) and in the highlands of Papua New Guinea (9.5%) (*3*). High mortality rates during influenza outbreaks in the developing setting have been ascribed to a lack of access to antimicrobial drugs to treat cases of secondary pneumonia and lack of access to health care in general (*1*).

Diarrheal disease is a major cause of illness and death throughout the world, with diarrheal outbreaks causing a substantial proportion of deaths (*4*). Endemic shigellosis is responsible for ≈10% of all cases of diarrhea among children <5 years of age living in developing countries and up to 75% of diarrheal deaths (*5**,**6*). Although epidemic *Shigella dysenteriae* causes the most dramatic form of *Shigella* spp. infections in developing countries with high attack rates and mortality rates, approximately half of the *Shigella* spp. infections are caused by endemic *Shigella* spp (*4*). Despite the endemicity of both influenza viruses and *Shigella* spp. in developing countries, data on their co-infection are lacking.

In mid-August 2009, an outbreak of bloody diarrhea and influenza-like illness (ILI) was reported to health authorities in Menyamya, a remote highland region of Morobe Province, with an estimated population of 10,000 persons. On August 28, an investigation was conducted to identify the cause and extent and to implement control measures.

Two sets of data were collected at the Hakwange Aid Post in Menyamya: 1) laboratory-investigated cases, 2) verbal autopsies. An additional dataset of clinical cases was subsequently collected from surrounding facilities in the district.

Rapid verbal autopsies were conducted by using standardized questionnaires. Bloody diarrhea was defined as acute onset of fever and diarrhea with visible blood in the stool. ILI was defined as acute onset of fever with cough or sore throat or both. Twenty deaths were identified in the Hakwange Aid Post catchment area, of which 11 were associated with bloody diarrhea and 9 with respiratory illness. Molecular methods were used to identify and characterize respiratory pathogens, and sequencing was used to identify genes that conferred enhanced pathogenicity. Influenza A virus was identified in 14 of 20 respiratory samples collected, of which 10 were subtyped as H3N2; the virus was A/Perth/16/09-like. During the investigation, patients with ILI were given oseltamivir.

Rectal swab specimens were transported in Cary-Blair media and were cultured within hours before serologic and biochemical testing were performed. Antimicrobial drug resistance testing was performed by using the Kirby-Bauer method. *S. flexneri* serotype 3 was isolated in 3 of 14 investigated cases of bloody diarrhea, with no other pathogens identified. *Shigella* spp. were resistant to amoxicillin, chloramphenicol, and co-trimoxazole but susceptible to ciprofloxacin. Patients received co-trimoxazole and, following sensitivity test results, ciprofloxacin or norfloxacin. Community health education sessions were conducted, and soap, jerry cans, and Aquatabs (Medentech Ltd, Wexford, Ireland) were distributed to households.

Early detection and intervention in disease outbreaks enable timely public health measures and may limit illness and death (*7*). Twenty deaths had already occurred in this provincial border community before our assessment, and an additional 200 deaths were associated with these conditions in neighboring provinces (*8*). The delayed reporting of these events from extremely isolated areas resulted in a delayed and less effective response. Although dealing with an outbreak is extremely challenging in this setting, strengthening the system for reporting such events from the district level has the potential to save lives.

Despite the high number of deaths associated with this outbreak of seasonal influenza A (H3), phylogenic analysis showed that the strain was similar to the low pathogenicity seasonal influenza virus that had circulated in the region during the previous 12 months. In our assessment, only 29% of those who sought treatment for respiratory symptoms and difficulty breathing were given antimicrobial drugs. The facility-based case-fatality ratios suggested a greater likelihood of death associated with possible co-infection (odds ratio 2.1, 95% confidence interval 0.5–7.4) (Table), but the difference was not significant. The major limitation of this investigation is the lack of microbiologic confirmation to allow wider assumptions to be made about possible co-infections, their effects (if any), and the role of other pathogens that cause similar clinical features.

**Table:** Descriptive epidemiology of concurrent outbreaks of bloody diarrhea and influenza-like illness, Menyamya District, Papua New Guinea, 2009

Variable	No. (%) patients*				
	Bloody diarrhea, n = 50	Influenza-like illness, n = 431	Possible co-infection, n = 131	Nonfebrile respiratory illness, n = 92	Total, n = 704
Aid post					
Hakwange	25 (50.0)	256 (59.4)	50 (38.2)	67 (72.8)	398 (56.5)
Kome	10 (20.0)	64 (14.9)	27 (20.6)	12 (13.0)	113 (16.1)
Kulolonguli	15 (30.0)	111 (25.8)	54 (41.2)	13 (14.1)	193 (27.4)
Male sex	24 (48.0)	206 (47.8)	71 (54.2)	40 (43.5)	341 (48.4)
Age group, y					
<5	11 (22.0)	118 (27.4)	33 (25.2)	10 (10.9)	172 (24.4)
5–14	14 (28.0)	103 (23.9)	23 (17.6)	22 (23.9)	162 (23.0)
15–44	21 (42.0)	169 (39.2)	58 (44.3)	50 (54.4)	298 (42.3)
>45	4 (8.0)	39 (9.1)	17 (13.0)	8 (8.7)	68 (9.7)
Unknown	0	2 (0.5)	0	2 (2.2)	4 (0.6)
Date of onset					
June	0	6 (1.4)	3 (2.3)	2 (2.2)	11 (1.6)
July	9 (18.0)	36 (8.4)	21 (16.0)	7 (7.6)	73 (10.4)
August	29 (58.0)	294 (68.2)	76 (58.0)	57 (62.0)	456 (64.8)
Unknown	12 (24.0)	95 (22.0)	31 (23.7)	26 (28.3)	164 (23.3)
Died	1 (2.0)	8 (1.9)	5 (3.8)	2 (2.2)	16 (2.3)

*Categories are mutually exclusive.

Ciprofloxacin is now recommended as the drug of choice for all patients with bloody diarrhea, regardless of their age (*9*). *Shigella* spp. have widespread resistance to the recommended treatment for bloody diarrhea in Papua New Guinea, co-trimoxazole, and no resistance to ciprofloxacin. This outbreak strain was resistant to co-trimoxazole, and its administration would have contributed little to limiting disease and its subsequent transmission. In the context of widespread illness and death, possibly associated with multidrug-resistant *Shigella* spp., a review of the national policy for the management of bloody diarrhea is urgently needed.
